# The impact of national cultural framework on college students’ willingness to communicate in oral English tasks in flipped classroom mode: a quasi experimental study

**DOI:** 10.3389/fpsyg.2026.1910813

**Published:** 2026-10-07

**Authors:** Pingting He

**Affiliations:** College of Foreign Languages, Hezhou University, Hezhou, China

**Keywords:** flipped classroom, national cultural framework, quasi experimental study, spoken English, willingness to communicate

## Abstract

Against the backdrop of “telling Chinese stories well in English,” a core value orientation of college English education in the new era, college oral English teaching undertakes the dual mission of fostering language proficiency and shaping cultural confidence. This study takes the flipped classroom model as the implementation carrier, constructs a national cultural framework with three dimensions of cultural cognition, cultural expression and cultural identity, and adopts a non-equivalent control group pretest-posttest quasi-experimental design to explore its effects and mechanisms on college students’ oral English willingness to communicate (WTC). Participants include 86 non-English major freshmen from a local comprehensive university in East China, divided into an experimental group (*n* = 43) receiving flipped classroom instruction integrated with the national cultural framework, and a control group (*n* = 43) receiving conventional themed flipped classroom teaching. The full intervention cycle lasts 16 weeks. Data are collected through oral English proficiency tests, L2 WTC scales, national culture framework scales, structured classroom observations and semi-structured interviews. Quantitative data are statistically analyzed with SPSS 26.0, and qualitative data are summarized via thematic coding. Results show that the experimental group achieves significantly higher post-test WTC scores with a large effect size. Cultural expression and cultural identity exert significant positive predictive effects on WTC, while the direct predictive effect of cultural cognition is not significant. Interview themes suggest reduced oral anxiety, enhanced self-efficacy and greater intrinsic motivation as possible explanations for these associations; mediation pathways were not statistically tested. This study verifies the facilitative effect of local cultural content on L2 WTC, providing replicable practical paradigms and empirical evidence for the ideological and political construction of college English courses and oral English teaching reform.

## 1 Introduction

With the continuous promotion of the construction of China’s international communication ability, “telling China’s story in English” has become the core value orientation of College English Teaching in the new era. However, two structural dilemmas persist in college oral English teaching. First, the “dumb English” phenomenon remains largely unaddressed: students’ overall oral English willingness to communicate (WTC) stays at a low level, with active in-class participation rates consistently below 30% as evidenced by multiple domestic empirical surveys (Baig and Yadegaridehkordi, 2023; Makena and Feni, 2023), and the imbalance between language input and output has not been effectively reversed. Second, teaching content shows a notable “Western cultural centralism” tendency. The long-term lack of national cultural elements has resulted in students’ “Chinese cultural aphasia”: while students can discuss Western topics smoothly, they find it difficult to accurately express the connotations of local culture and often encounter expressive obstacles in cross-cultural communication (Spaic et al., 2025).

Flipped classroom releases sufficient interactive space for oral teaching through the inverted process of “knowledge transfer before class+knowledge internalization in class”. Previous studies have confirmed that it can improve oral fluency and reduce language anxiety (Katona and Gyonyoru, 2025). However, the existing research focuses on the technical optimization of the teaching process, and generally ignores the deep impact of the cultural attribute of the teaching content on the psychological level of learners (Huang et al., 2023). The cultural familiarity and emotional relevance of oral tasks have a direct impact on learners’ expression desire and engagement. However, whether the embedding of local cultural content can fundamentally stimulate the expression power and stably improve the willingness to communicate is still lacking of strict experimental evidence and systematic mechanism interpretation (Egara and Mosimege, 2024). Given that communicative intention is the core antecedent variable to predict the actual second language production behavior, this study embedded the national cultural framework into the flipped classroom oral teaching, and tested its effect and path of action on College Students’ communicative intention through a 16 week quasi experiment (Lu et al., 2023).

A systematic review of existing literature identifies three core research gaps. First, flipped classroom oral teaching research prioritizes linguistic skill development over cultural dimensions, and rarely addresses the impact of cultural integration on affective variables such as WTC (Ray and Sikdar, 2024). Second, L2 WTC studies predominantly focus on individual psychological factors, while empirical investigation of local culture as a socio-situational predictor remains insufficient (Qi et al., 2024; Sada et al., 2023). Third, research on national culture integration is mostly based on experience summaries and lesson design, lacking long-term controlled experimental evidence and in-depth exploration of the “cognition–expression–willingness” mechanism chain (Apat et al., 2023; Al Awlaqi and Ghozali, 2023).

## 2 Literature review and theoretical basis

### 2.1 Flipped classroom and oral English teaching

Domestic empirical research shows that the flipped classroom model can significantly improve students’ oral fluency and language complexity, increase the effective interaction time of the classroom by 50% compared with the traditional classroom, and effectively reduce the level of Oral Anxiety and improve classroom participation (Musliadi et al., 2024; Wang et al., 2024; Alkhaldi et al., 2023). The structural strengths of flipped classrooms fit well with the core demands of oral English teaching. Pre-class knowledge transfer frees up in-class time from mechanical knowledge explanation, reserving abundant time for high-density oral output, peer negotiation and interactive feedback that oral competence development heavily relies on. Out-of-class preview also allows learners to digest basic language points at their own pace, lowering cognitive pressure so that in-class activities can focus on meaning-oriented communication rather than rote drills.

However, the existing research has obvious content level limitations: most of the flipped classroom is regarded as a simple innovation in the form of teaching organization, the topic of oral task is mostly the topic of western culture in the textbook, and the educational value and motivation potential at the content level are not fully tapped (Diobi and Nashruddin, 2023; Setiawan et al., 2023). Even with sufficient interactive time, when oral tasks centre on unfamiliar Western cultural themes, learners often struggle with insufficient background schemata. Content-related obstacles may override the structural advantages of flipped design, triggering expression barriers and dampening learners’ willingness-to-communicate. This practical dilemma constitutes the core motivation for embedding national cultural content into flipped oral teaching in this study (Lemana et al., 2023; Vaishnav, 2024).

### 2.2 Theoretical evolution and localization of L2 communicative intention

The concept of willingness to communicate originated from the field of mother tongue communication research, and is defined as the psychological tendency of individuals to actively choose to participate in communication in a specific situation (Sawalmeh and Dey, 2023). Subsequently, MacIntyre et al. (1998) systematically introduced the construct into second language acquisition research and proposed the classic pyramid heuristic model, which has served as the foundational theoretical framework in this field ever since (MacIntyre et al., 1998). The model hierarchically organizes predictors of WTC into six layers from bottom to top: personality factors, emotional-cognitive factors, motivational factors, situational factors, communicator status, and willingness to communicate itself. The core view is that the closer to the top of the pyramid, the more direct the impact of factors on communication willingness. Social culture and situational factors play an indirect role through multi-layer intermediary chain.

Domestic localization research shows that Chinese college students’ willingness to communicate in English as a whole is at the lower middle level, and their willingness to speak publicly in class is significantly lower than that of group discussion. Topic familiarity is an important situational factor (Makena and Feni, 2023). When discussing familiar local life topics, the fluency and initiative of students’ expression will be significantly improved (Castro, 2023). However, at present, few studies have systematically discussed the independent impact of national culture, a special topic type, on the willingness to communicate, and there is also a lack of causal verification through long-term intervention experiments, which constitutes the core research space of this study (Zheng et al., 2026).

### 2.3 Research context of integrating national culture into English teaching

The phenomenon of “Chinese Cultural Aphasia” has been widely concerned by the academic community since it was proposed at the beginning of this century. It means that English learners have a certain language foundation, but they are unable to accurately and appropriately express the content of Chinese local culture in English. This local challenge resonates with broader international second-language acquisition scholarship: global EFL research has long documented that learners struggle to articulate their own heritage-culture content in target-language classrooms, a shared challenge observed across many non-English-dominant contexts (Chen, 2025; Liang et al., 2026). Internationally, WTC scholarship has highlighted that culturally-connected, identity-relevant topics boost learners’ communicative inclination, yet most empirical work focuses on immigrant or minority-learner populations, with far fewer classroom-based quasi-experiments centring on mainstream learners’ own national-cultural resources (Hu and Wang, 2023; Lee and Taylor, 2024).

For more than 20 years, the Chinese academic community has generally called for the integration of the Chinese cultural system into English teaching to achieve the organic unity of language instrumentality and cultural humanism. The existing domestic research is mainly divided into three directions: first, it demonstrates the necessity and time value of integration from the perspectives of national cultural security, cultural self-confidence cultivation, and cross-cultural communicative competence cultivation (Zhi and Wang, 2024); The second is to build a multi-level teaching system including traditional ideas, folk culture, Intangible Cultural Heritage Inheritance and other contents; The third is to explore the practical path of cultural integration through lesson research (Li et al., 2025; Lee and Taylor, 2024).

In the field of oral English teaching, preliminary domestic studies have shown that cultural familiarity can reduce learners’ cognitive load, enable them to allocate more attention to language expression, and then improve output fluency; Cultural identity can stimulate intrinsic motivation for expression, and promote oral output from “passively completing tasks” to “actively transmitting views” (Shafiee Rad, 2024; Yunus et al., 2023). Parallel international studies on heritage-related content integration yield similar correlational findings, though few combine structured national-cultural frameworks with flipped-classroom oral interventions to trace causal-like effects on L2 WTC (Fisher et al., 2024). However, most of the existing researches are short-term and scattered teaching attempts, lack of systematic theoretical framework and strict effect evaluation, especially fail to reveal the deep psychological mechanism of national culture affecting communication willingness, and there is still room for improvement in the systematicness and persuasiveness of the research.

### 2.4 Core connotation and dimension construction of National Cultural Framework

Grounded in social identity theory and communicative competence frameworks, and aligned with the practical needs of oral English teaching, this study conceptualizes the “national cultural framework” and operationalizes it into three progressive dimensions. The cultural cognitive dimension captures learners’ mastery of core knowledge of Chinese traditional culture, including historical allusions, traditional customs, cultural symbols, and core values, serving as the knowledge foundation of the framework (Fisher et al., 2024). The cultural expression dimension refers to learners’ abilities and strategies to accurately articulate national cultural content in English, such as translating cultural proper nouns, interpreting cultural concepts, and comparing cross-cultural differences, acting as the critical bridge between cognition and communicative behavior (Soyoof, 2023).

The cultural identity dimension reflects learners’ emotional attitudes and value identification with their national culture, including cultural pride, inheritance awareness, and dissemination willingness, and constitutes the emotional core and motivational driver of the framework (Alrabai, 2024; Lee and Lu, 2023). Aligned with Bandura’s self-efficacy perspective, these three dimensions operate sequentially across knowledge, ability, and affective levels, comprehensively capturing the multiple objectives of integrating national culture into oral English instruction.

The dimension of cultural identity refers to learners’ emotional attitude and value identity towards their own national culture, including cultural pride, awareness of inheritance, and willingness to spread. It is the emotional core and power source of the framework (Saimon et al., 2023; Al-Obaydi et al., 2023). The three dimensions are progressive layer by layer, covering three levels of knowledge, ability and emotion, which can comprehensively reflect the multiple goals of integrating national culture into oral English teaching (Cornejo and Kam, 2024). Based on the existing literature review and the actual needs of oral teaching, this study constructs the core concept of “national cultural framework” and operationalizes it into three progressive dimensions.

### 2.5 Theoretical framework integration

Based on the flipped classroom teaching theory, the pyramid model of second language communication willingness and the related concepts of cultural education, this study constructs an integrated research theoretical framework.

This integrated framework uses the flipped classroom as the instructional setting and organizes national cultural content into cultural cognition, cultural expression and cultural identity. Oral willingness to communicate (WTC), assessed across in-class speaking, group discussion, peer communication and public presentation, is the primary outcome. Three distinct empirical questions concern between-group WTC outcomes (H1), zero-order associations between the cultural dimensions and WTC (H2), and their unique statistical contributions in a simultaneous regression model (H3). Oral anxiety, self-efficacy and intrinsic motivation provide possible theoretical explanations that are explored through interview themes; they are not measured mediators in the reported quantitative models.

Accordingly, the study asks whether the intervention is associated with higher WTC and greater WTC gains, whether each cultural dimension is positively correlated with WTC, and whether each dimension predicts WTC when the other two dimensions are held constant. Three main hypotheses and eight sub-hypotheses are stated below. The qualitative inquiry separately explores students’ accounts of possible mechanisms and is not treated as an additional statistical hypothesis test.

*H1*: Flipped classroom instruction integrated with the national cultural framework produces more favorable WTC outcomes than conventional themed flipped classroom instruction.

*H1a*: The experimental group has higher post-test WTC total and sub-dimension scores than the control group.

*H1b*: The experimental group shows a significantly greater pretest-to-posttest increase in WTC than the control group.

*H2*: Each dimension of the national cultural framework is positively correlated with oral English WTC.

*H2a*: Cultural cognition is positively correlated with WTC.

*H2b*: Cultural expression is positively correlated with WTC.

*H2c*: Cultural identity is positively correlated with WTC.

*H3*: The three cultural dimensions make positive unique contributions to post-test WTC in a simultaneous multiple regression model. The dimension-specific predictions are separated as follows.

*H3a*: Cultural cognition positively predicts WTC after controlling for cultural expression and cultural identity.

*H3b*: Cultural expression positively predicts WTC after controlling for cultural cognition and cultural identity. The additional comparative expectation is that cultural expression has the strongest positive standardized coefficient among the three predictors.

*H3c*: Cultural identity positively predicts WTC after controlling for cultural cognition and cultural expression.

For H3, prediction refers to a conditional statistical association in the experimental group’s post-test data, rather than a causal effect or out-of-sample prediction. The positive-coefficient component of H3b and its comparative component require separate evaluation: a significant coefficient tests the former, whereas coefficient ordering alone does not establish that one predictor is statistically stronger than another. The original comparative expectation is retained, but no formal coefficient-difference test is reported.

## 3 Research design

### 3.1 Research design type

In this study, the non-equivalent control group was used as the pre and post calibration experimental design. Two complete natural teaching classes were selected as the experimental group and the control group, respectively. Before the experiment, the two groups of students’ oral level and communicative intention were tested to verify the consistency of the baseline level; The experimental group received the flipped classroom oral teaching integrated with the national cultural framework, while the control group received the flipped classroom teaching with the conventional theme; After the complete intervention cycle, the post test was conducted, and the net effect of teaching intervention was inferred by comparing the post-test differences between the two groups. The choice of quasi experimental design rather than strict randomized experiment is mainly due to the ecological validity of teaching research. The teaching arrangement of natural class is more in line with the real teaching situation, and the practical reference value of the research conclusion is higher. At the same time, the design of pre-test and post-test can also better control the internal validity threats such as maturity and history.

To visually clarify the overall research arrangement, this study constructs a quasi experimental design framework, as shown in Figure 1. The whole research follows the logical sequence of pre-test → intervention → post-test. Two intact natural classes were divided into experimental group and control group without random grouping. The pre-test ensured the baseline homogeneity of the two groups, the 16-week teaching intervention was the core operation of the study, and the post-test data was the main basis for judging the intervention effect. This design effectively controls internal validity threats such as maturity effect and historical interference, and conforms to the ecological characteristics of real college English classroom teaching.

**Figure 1:**
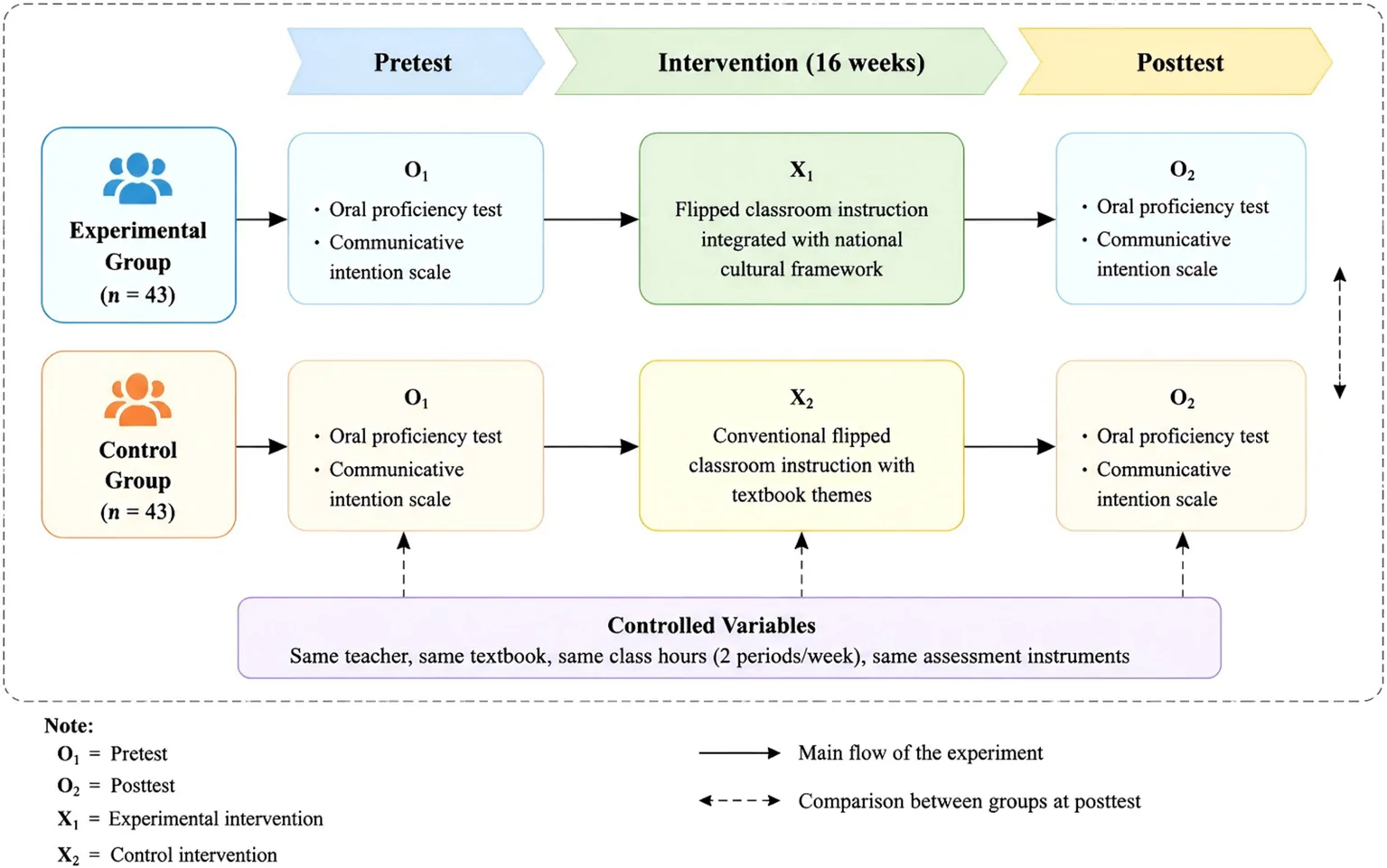
Framework of the non-equivalent control group pretest-posttest quasi-experimental design.

### 3.2 Research object

This study uses convenience sampling method to select students from one or two natural classes of non English Majors of grade 2024 in a local comprehensive university in East China as the research object, covering different professional backgrounds such as business and engineering. The study set three inclusion criteria: firstly, no long-term overseas life experience; secondly, no minor in English related professional courses; thirdly, voluntary participation in the study and the signing of informed consent. There was no loss of subjects during the experiment, and the final effective sample was 86, 43 in the experimental group and 43 in the control group. All research data are strictly confidential and only used for academic statistical analysis. The demographic information and baseline data of the two groups are shown in Table 1.

**Table 1:** Demographic information and baseline data of subjects.

Variable	Category	Experimental group (*n* = 43)	Control group (*n* = 43)	Total (*n* = 86)	Inter group test *p*-value
Gender	Male	19 (44.2%)	21 (48.8%)	40 (46.5%)	0.673
	Female	24 (55.8%)	22 (51.2%)	46 (53.5%)	
Age	18 years old	28 (65.1%)	26 (60.5%)	54 (62.8%)	0.825
	19 years old	13 (30.2%)	15 (34.9%)	28 (32.6%)	
	≥ 20 years old	2 (4.7%)	2 (4.7%)	4 (4.7%)	
College entrance examination English scores	M ± SD	112.35 ± 9.62	111.87 ± 10.14	112.11 ± 9.83	0.824
Oral proficiency pre test	M ± SD	62.47 ± 8.35	61.93 ± 9.12	62.20 ± 8.74	0.775
Communicative intention pretest	M ± SD	2.98 ± 0.54	2.97 ± 0.57	2.97 ± 0.55	0.935

It can be seen from the data in the table that the differences between the two groups in gender composition, age distribution, college entrance examination English scores, oral pre-test scores, and communication willingness pre-test did not reach a statistically significant level, and the baseline level was highly consistent, meeting the homogeneity requirements of quasi experimental research. In order to further rigorously verify the inter group balance of oral proficiency, independent sample t-test was conducted on the pre-test scores of oral proficiency. The specific results are shown in Table 2.

**Table 2:** Independent sample *t*-test of oral English pretest in experimental group and control group.

Group	*N*	Mean value	Standard deviation	Levene variance homogeneity test p	t value	df	*p* value
Experimental group	43	62.47	8.35	0.612	0.287	84	0.775
Control group	43	61.93	9.12	—	—	—	—

Levene variance homogeneity test showed that the *p* value was greater than 0.05, which met the variance homogeneity hypothesis, so the combined variance was used to calculate the T value. The final results showed that there was no significant difference in the pre-test scores between the two groups, the experimental baseline was stable, and the differences in the post-test could be attributed to the role of teaching intervention.

### 3.3 Research tools

In this study, quantitative and qualitative research tools are comprehensively used to realize the triangular cross validation of results. The oral English proficiency test adopts a combination of impromptu speech and situational dialogue, with a full score of 100. In the impromptu speech part, the students are required to express themselves in 2 minutes around the designated theme, and in the situational dialogue part, they are required to interact in groups of two for 3 minutes according to the set scene. The scoring criteria cover five dimensions: pronunciation and intonation, vocabulary and grammar, fluency, content richness, and communicative appropriateness, with 20 points for each dimension. The test was conducted by two teachers with more than 5 years’ oral teaching experience. Importantly, raters were kept blind to group assignment: all participant identifiers and group-related contextual cues were stripped from oral recordings to minimize rater bias stemming from visible differences in task content across groups. The rater’s reliability ICC was 0.86, reaching a good level. The final score was taken as the average of the two teachers’ scores.

The second language communicative intention scale is revised based on the second language classroom communicative intention scale compiled by Peng in 2007 and combined with the actual situation of College English classroom in China. The scale consists of 18 items, which are divided into four sub dimensions: willingness to speak in class, willingness to discuss in groups, willingness to communicate with peers, and willingness to show to the public. Likert 5-point scoring method is adopted in the scale. 1 means completely unqualified, and 5 means fully qualified. The higher the score, the stronger the willingness to communicate. The pre survey data showed that the kmo value of the scale was 0.872, the Bartlett ball test reached a significant level, and the structural validity was good. The internal consistency reliability coefficient of each dimension is shown in Table 3.

**Table 3:** Reliability coefficient of second language communication willingness scale.

dimension	Number of items	Cronbach’sα
Willingness to speak in class	5	0.823
Willingness to discuss in groups	4	0.796
Willingness to communicate with peers	5	0.841
Public display willingness	4	0.815
Total quantity table	18	0.897

The Cronbach’s α coefficients of each dimension and total scale are visualized in Figure 2. It can be seen that the reliability coefficients of all sub-dimensions are above 0.78, and the total reliability of the two core scales exceeds 0.89. Combined with the KMO value and Bartlett test results mentioned above, all research tools have good internal consistency reliability and structural validity, which can ensure the stability and credibility of the collected research data.

**Figure 2:**
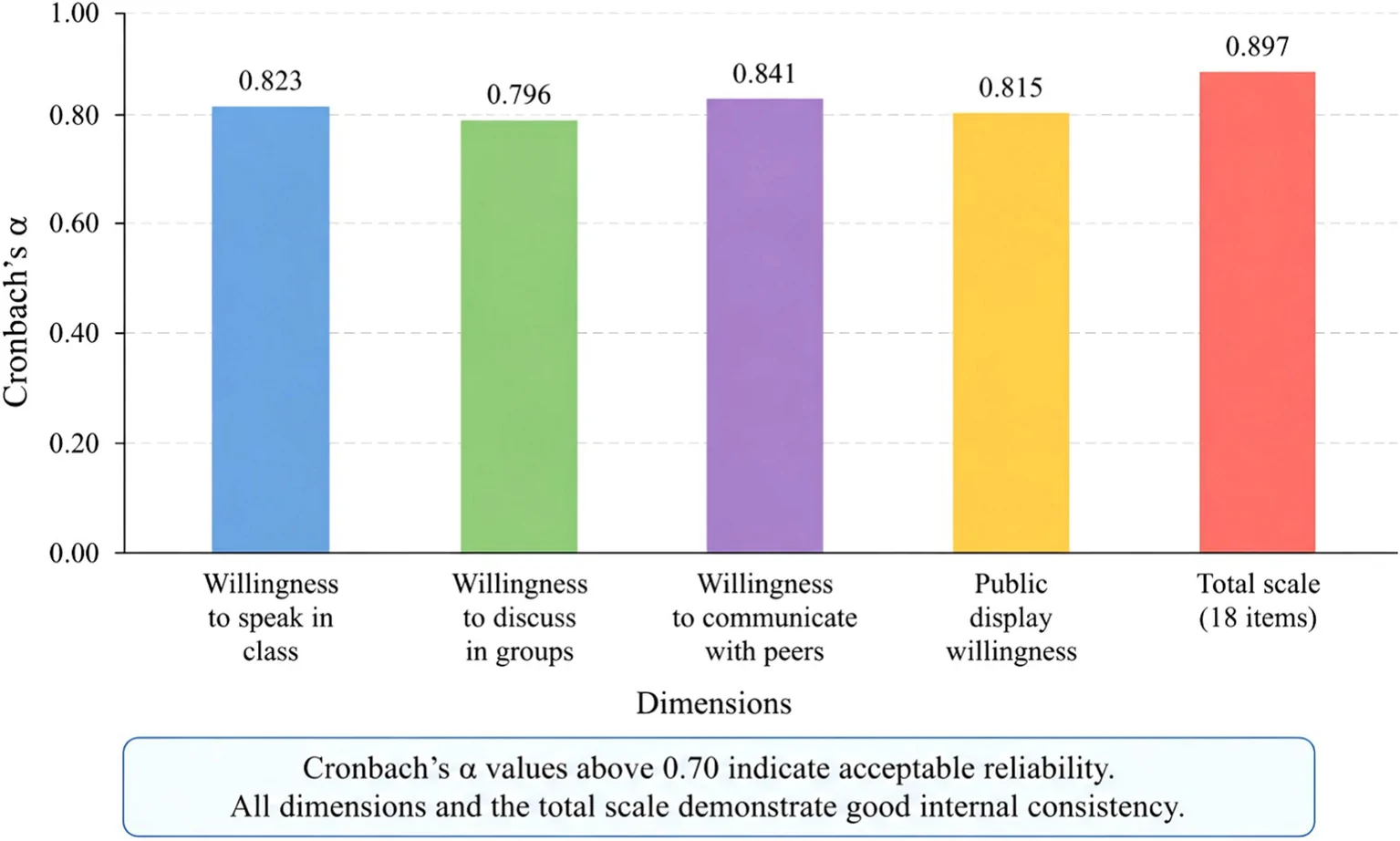
Reliability coefficients of all research scales.

The national culture framework scale is self-developed based on the three-dimensional framework constructed in this study, and contains 21 items. Among them, 7 items of cultural cognition dimension mainly measure students’ mastery of traditional cultural knowledge; Cultural expression dimension 7 questions, mainly measuring students’ self-confidence and ability perception in expressing cultural content in English; Cultural identity dimension 7 questions, mainly measuring students’ cultural emotional attitude and communication willingness. Likert 5-point scoring was also used for the scale. The pre survey results showed that the kmo value of the scale was 0.845, Bartlett test was significant, exploratory factor analysis extracted three common factors, and the cumulative variance interpretation rate was 62.37%, which met the research requirements. The internal consistency reliability coefficient of each dimension is shown in Table 4. The full item sets for both the revised L2 WTC scale and self-developed national cultural framework scale, as well as the semi-structured interview outline, are presented in (Supplementary Appendix A–C) for research replication purposes.

**Table 4:** Reliability test results of national culture framework scale.

Dimension	Number of items	Cronbach’s *α*
Cultural Cognition	7	0.784
Cultural expression	7	0.832
cultural identity	7	0.856
Total quantity table	21	0.902

Classroom observation and semi-structured interview are used as qualitative research tools to supplement and explain quantitative research results. During the experiment, a total of 8 non participatory observations were conducted in the classroom, which were arranged in the second, fourth, sixth, eighth, tenth, twelfth, fourteenth and sixteenth weeks of the experimental cycle. The structured observation scale was used to record the number of students’ active speeches in class, the length of group discussion participation, and the frequency of classroom presentation and interaction. Finally, the average value of the class was taken for statistical analysis. After the experiment, 12 students from the experimental group were selected by purposive sampling method for semi-structured interviews. Four students were selected from each group according to the level of high, medium and low willingness to communicate. The interview outline was designed around three core themes: the feeling of cultural integration into teaching, the change and reasons of willingness to communicate, and suggestions for teaching improvement. The duration of each interview is limited to 15 to 20 min. The whole process is recorded and transcribed into text, and the final transcribed text is about 32,000 words.

### 3.4 Experimental implementation process

The complete cycle of this experiment is 16 weeks, corresponding to the second semester of the 2024–2025 academic year, with 2 class hours per week, a total of 32 class hours. Both classes adopt the flipped classroom teaching mode, use the same set of College English core textbooks, and are taught by the same teacher. The interference of irrelevant variables such as teacher factors and class hour factors is strictly controlled. The only difference between the two groups is the content design of the oral task. The oral task of the experimental group is integrated into the national cultural framework, and the oral task of the control group uses the original common theme of the textbook. The experimental group adopted the three-stage flipped teaching process of “cultural input before class—cultural interaction in class—cultural development after class”. The overall process is shown in Figure 3.

**Figure 3:**
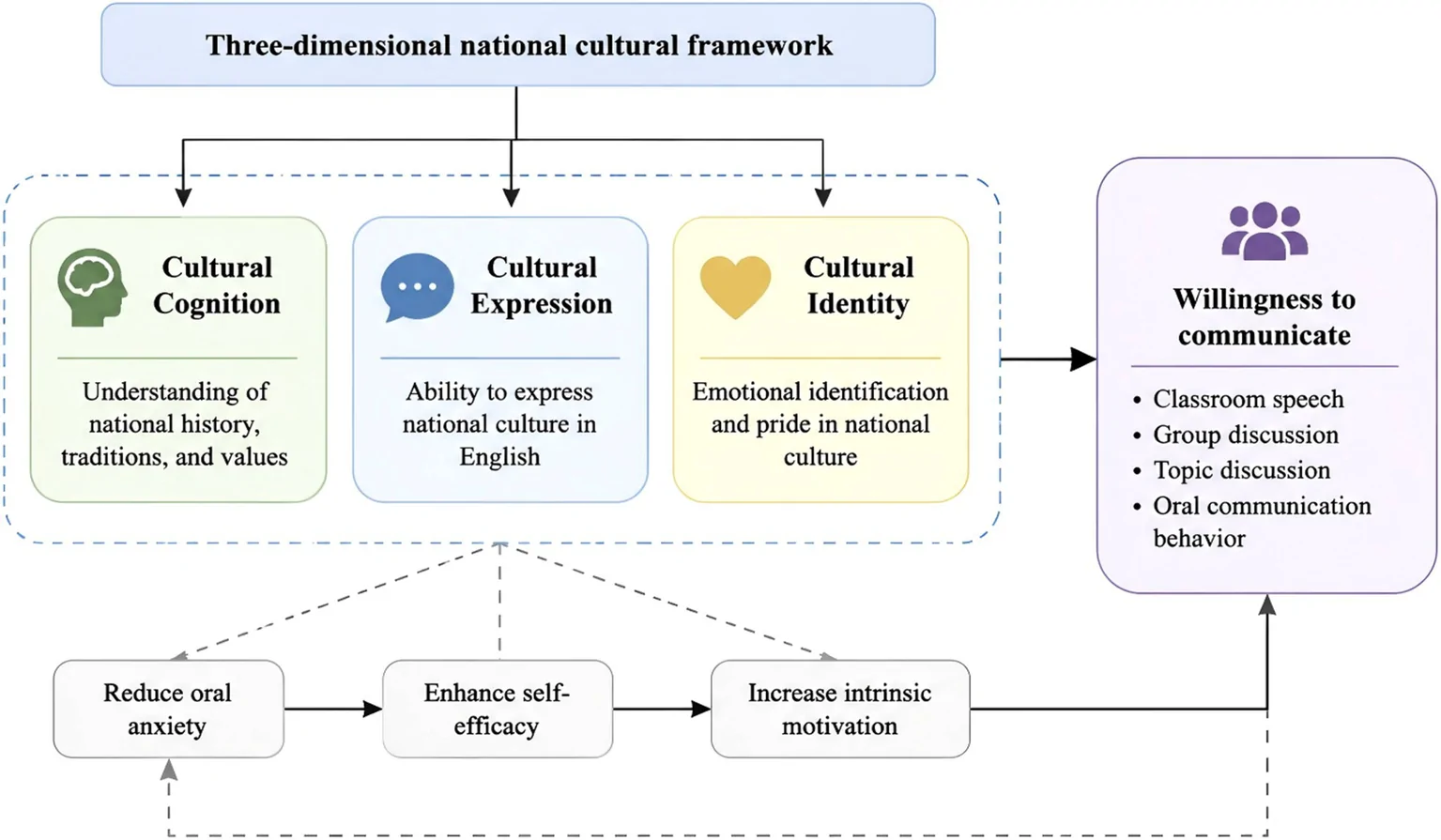
Flipped classroom teaching flow chart integrated into the framework of national culture.

The experimental group adopts an exclusive teaching mode of “pre class cultural input—in class cultural interaction—post class cultural expansion”. Before class, students rely on Chaoxing Learning Platform to complete independent learning, watching 8-min language knowledge micro lessons and 5-min ethnic culture English micro lessons. The curriculum includes 8 gradient cultural units, including traditional festivals and Chinese cuisine, with content transitioning from concrete life culture to abstract ideological culture. Students are required to complete online testing and record English oral assignments to elaborate on cultural perspectives. Based on this, teachers can organize student questions and clarify classroom priorities.

The 70 min class is divided into four parts: rapid testing, group cultural exploration, achievement display, and feedback summary. All discussion and presentation tasks revolve around the theme of Chinese ethnic culture, and English output exercises are carried out based on real communication scenarios. After class, students expand their learning by watching cultural English documentaries, writing oral reflection logs, and participating in the online forum “Telling Chinese Stories in English”, achieving the integration of cultural knowledge learning and application.

The control group followed the same flipped classroom process and classroom activities, watching accompanying language micro lessons and completing basic oral exercises before class, and conducting theme discussions and oral presentation activities during class. However, the entire teaching process of this group does not integrate ethnic cultural content. All oral tasks and classroom themes are universal themes of the textbook, including regular content such as campus life, environmental protection, technological development, and career planning. After class, only routine exercises are completed in accordance with the textbook, and there are no cultural expansion tasks.

The core difference between the two groups lies in the setting of oral task content and extended learning activities. The detailed comparison of teaching links is presented in Figure 4. The experimental group took national culture as the main line throughout the whole teaching process, forming a closed-loop learning of cultural input, interaction and output. By contrast, the control group adopted the traditional textbook topics without additional cultural expansion design. Except for the task content, all teaching conditions such as class hours, teachers and flipped classroom process were kept consistent, which guaranteed the single variable principle of the experiment.

**Figure 4:**
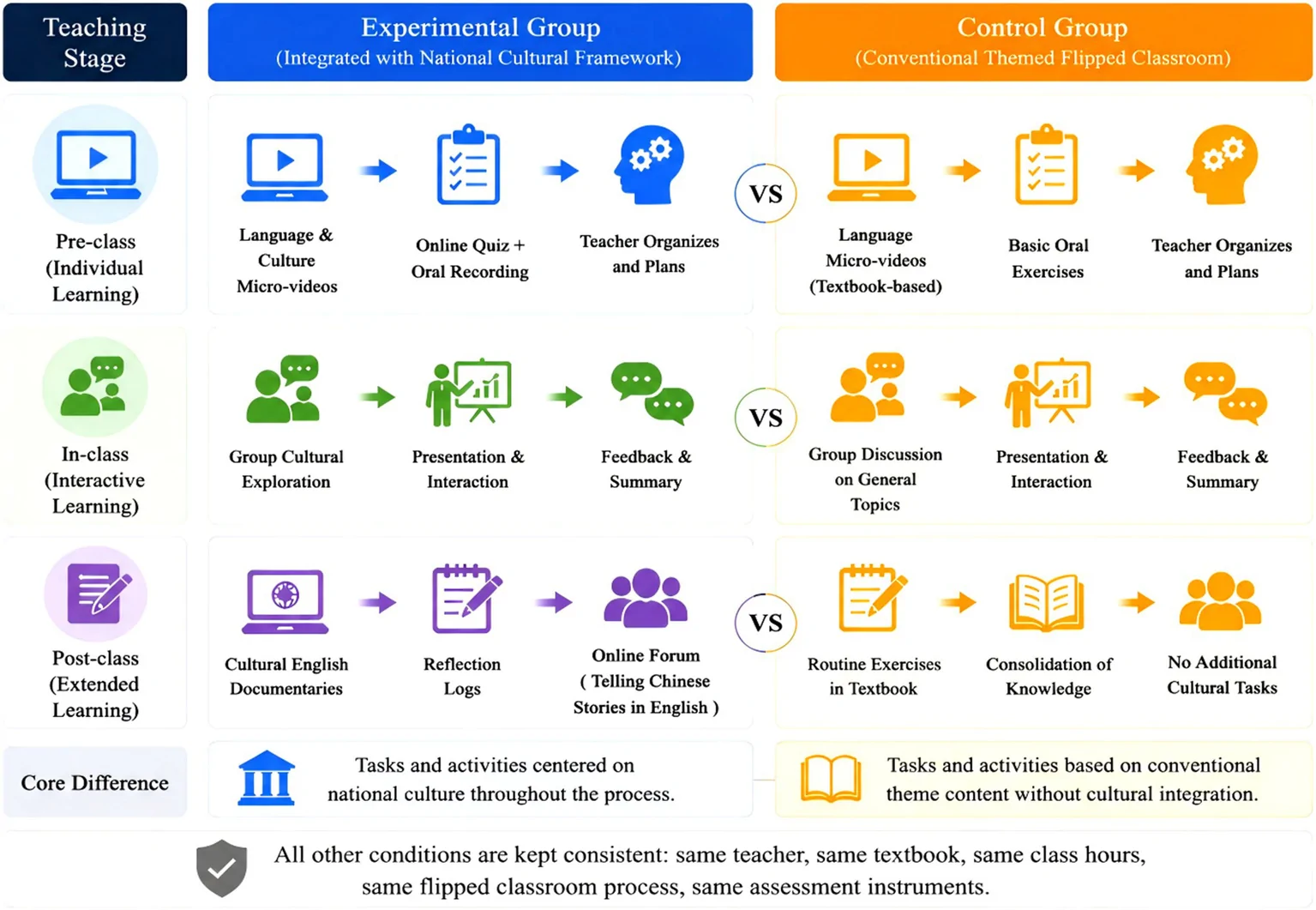
Comparison of teaching intervention content between experimental group and control group.

### 3.5 Data processing and analysis methods

The quantitative data of this study were processed and analyzed by SPSS 26.0 statistical software, and the qualitative data were analyzed by NVivo 12 software. The significance level of statistical analysis was uniformly set as *α* = 0.05. The core statistical methods include independent sample *t*-test, paired sample *t*-test, Pearson correlation analysis and multiple linear regression analysis. The independent sample *t*-test is used to compare the difference of the post-test mean between the experimental group and the control group, and its calculation formula is shown in Equation 1:

The analyses are mapped to the hypotheses in the same order as the results: H1a uses between-group post-test comparisons; H1b concerns between-group differences in pretest-to-posttest change; H2a-H2c use Pearson correlations; and H3a-H3c use the partial coefficients from one simultaneous regression with the three cultural dimensions entered together. Correlation and regression analyses use the experimental group’s post-test data (*n* = 43). The reported paired *t*-tests assess within-group change and do not by themselves test H1b. No direct between-group gain test, group-by-time interaction, formal comparison of regression coefficients, or mediation analysis is reported. Secondary oral-performance outcomes, classroom observations and interview findings are presented separately from these hypothesis-linked analyses.

$$t=\frac{{\bar{X}}_{1}-{\bar{X}}_{2}}{\sqrt{\frac{(n_{1}-1)s_{1}^{2}+(n_{2}-1)s_{2}^{2}}{n_{1}+n_{2}-2}}\cdot \sqrt{\frac{1}{n_{1}}+\frac{1}{n_{2}}}}$$ (1)

Where ${\bar{X}}_{1}$ and ${\bar{X}}_{2}$ are the sample mean values of the experimental group and the control group respectively, $S_{1}^{2}$ and $S_{2}^{2}$ are the sample variance of the two groups respectively, n1 and n2 are the sample sizes of the two groups respectively, and the degree of freedom $d_{f}=n_{1}+n_{2}-2$ .

Pearson correlation analysis is used to examine the linear correlation between the dimensions of the national cultural framework and the willingness to communicate. It calculation formula is presented in Equation 2:

$$r=\frac{\sum_{i=1}^{n}(x_{i}-\bar{x})(y_{i}-\bar{y})}{\sqrt{\sum_{i=1}^{n}{\left(x_{i}-\bar{x}\right)}^{2}}\cdot \sqrt{\sum_{i=1}^{n}{\left(y_{i}-\bar{y}\right)}^{2}}}$$ (2)

Where $x_{i}$ and $y_{i}$ are the individual values of the two variables respectively, $\bar{x}$ and $\bar{y}$ are the sample mean values of the two variables respectively, and n is the sample size.

Multiple linear regression analysis is used to test the predictive effect of various dimensions of the national cultural framework on communication willingness. The regression equation is defined in Equation 3:

$$Y=\beta_{0}+\beta_{1}X_{1}+\beta_{2}X_{2}+\beta_{3}X_{3}+\varepsilon$$ (3)

Where, Y is the total score of willingness to communicate, $\beta_{0}$ is a constant term, $\beta_{1}$ , $\beta_{2}$ and $\beta_{3}$ are non-standardized regression coefficients, $X_{1}$ , $X_{2}$ and $X_{3}$ represent the three dimensions of cultural cognition, cultural expression and cultural identity respectively, and ε is a random error term.

The qualitative data analysis follows the three-level coding process of open coding, spindle coding and selective coding. Two researchers independently encode the data, and the coding consistency coefficient reaches 89%. The codes with differences are agreed by both parties through discussion to ensure the reliability of the analysis results.

## 4 Research results

Results are organized sequentially by H1, H2 and H3. Section 4.1 presents baseline context and WTC outcomes relevant to H1a and H1b; Section 4.2 addresses the three correlations specified in H2; and Section 4.3 separates the regression evidence for H3a-H3c and summarizes the hypothesis evaluations. Section 4.4 is a standalone account of supplementary secondary outcomes and qualitative corroboration.

### 4.1 H1: intervention effects on willingness to communicate

#### 4.1.1 Baseline context for H1

Table 5 presents the pre-test WTC total and sub-dimension scores for the two groups. None of the reported between-group comparisons reached statistical significance (all *p* > 0.05). These results provide baseline context for H1, but non-significant tests do not establish group equivalence or remove possible confounding from the non-randomized assignment of two intact classes.

**Table 5:** Pre-test comparison of communication willingness between the experimental group and the control group.

Dimension	Experimental group (*n* = 43) m ± SD	Control group (*n* = 43) m ± SD	*t* value	*p* value
Willingness to speak in class	2.87 ± 0.65	2.91 ± 0.71	−0.276	0.783
Willingness to discuss in groups	3.12 ± 0.58	3.08 ± 0.63	0.309	0.758
Willingness to communicate with peers	3.35 ± 0.62	3.29 ± 0.67	0.431	0.667
Public display willingness	2.56 ± 0.74	2.61 ± 0.69	−0.328	0.744
Total score of willingness to communicate	2.98 ± 0.54	2.97 ± 0.57	0.082	0.935

#### 4.1.2 H1a: between-group post-test comparisons

After 16 weeks, the experimental group had a higher WTC total score than the control group (3.87 ± 0.46 versus 3.31 ± 0.52; reported *t* = 5.324, *p* < 0.001, Cohen’s d = 1.15; Table 6). All four WTC sub-dimensions were also higher in the experimental group (all reported *p* < 0.001), with reported effect sizes ranging from 0.85 to 1.10. The largest sub-dimension effect size was for group discussion (*d* = 1.10). Figure 5 illustrates the total-score difference. These post-test comparisons support H1a within the limitations of the two-class quasi-experimental design.

**Table 6:** Post-test comparison of communication willingness between the experimental group and the control group.

Dimension	Experimental group M ± SD	Control group M ± SD	*t* value	*p*-value	Cohen’s d
Willingness to speak in class	3.82 ± 0.57	3.24 ± 0.65	4.453	<0.001***	0.96
Willingness to discuss in groups	4.01 ± 0.49	3.42 ± 0.58	5.107	<0.001***	1.10
Willingness to communicate with peers	4.15 ± 0.53	3.67 ± 0.61	3.952	<0.001***	0.85
Public display willingness	3.48 ± 0.66	2.89 ± 0.72	3.986	<0.001***	0.86
Total score of willingness to communicate	3.87 ± 0.46	3.31 ± 0.52	5.324	<0.001***	1.15

***p < 0.001.

**Figure 5:**
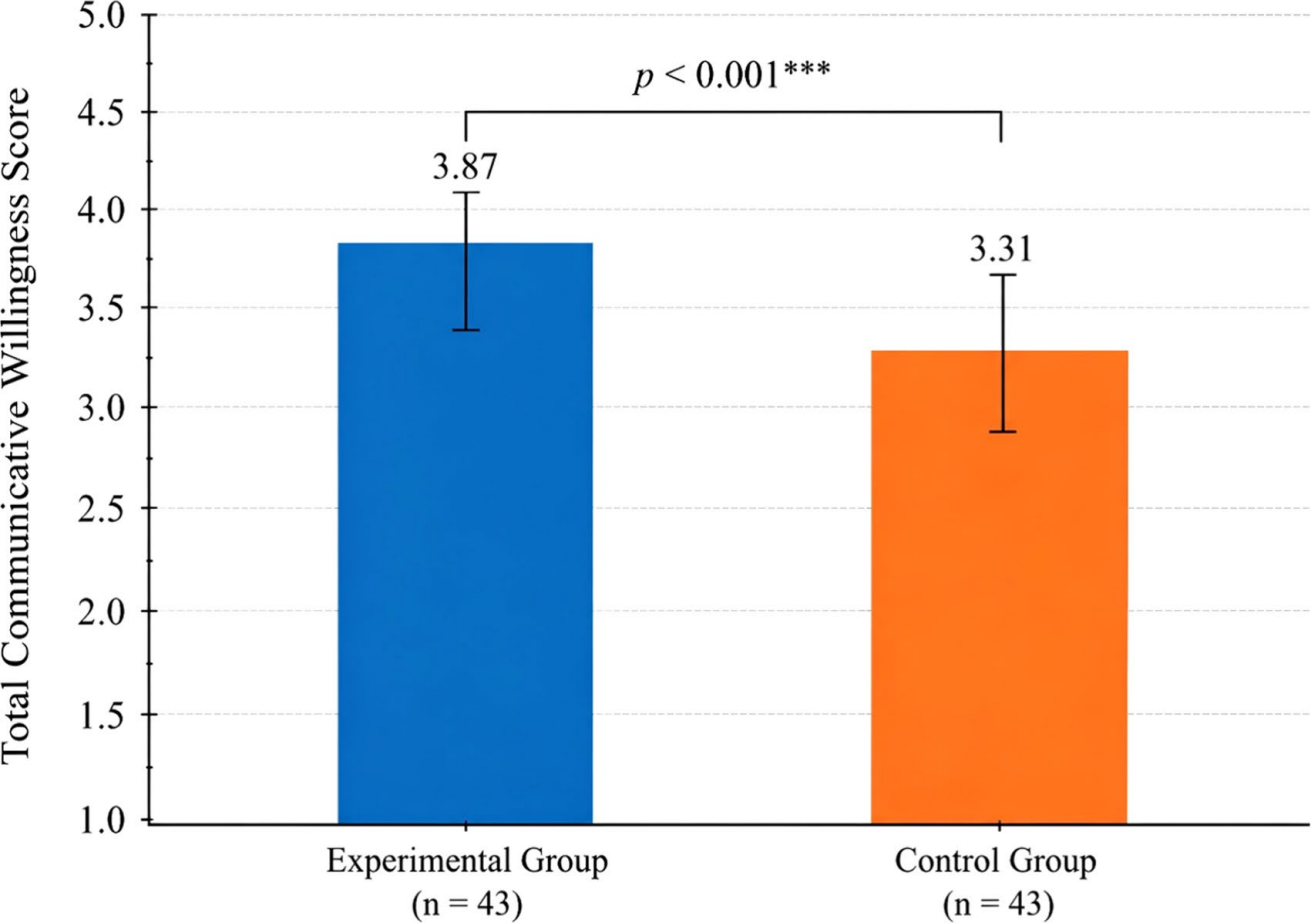
Histogram for comparison of the total score of communicative intention between the experimental group and the control group.

In order to visually present the overall difference between the two groups in the post-test, a comparison histogram of the total score of communicative intention is drawn, as shown in Figure 5. The figure clearly shows that the average total score of the experimental group is significantly higher than that of the control group, and the difference between the two groups is close to 0.6 points. The height difference of the histogram directly reflects the positive effect of the national cultural framework intervention.

#### 4.1.3 H1b: pretest-to-posttest changes

Within the experimental group, the reported paired t-tests indicated increases in the WTC total score and all four sub-dimensions (all *p* < 0.001; Table 7). The total-score gain was 0.89 points, from 2.98 to 3.87 (*t* = 11.568, df = 42). The largest sub-dimension gain was in willingness to speak in class (0.95 points). These tests establish within-group change; the hypothesis of a greater gain than in the control group is evaluated separately below.

**Table 7:** Paired sample *t*-test before and after the test of communicative intention in the experimental group.

Dimension	Pre-test m ± SD	Post-test m ± SD	Mean difference	*t* value	df	*p*-value
Willingness to speak in class	2.87 ± 0.65	3.82 ± 0.57	0.95	9.872	42	<0.001***
Willingness to discuss in groups	3.12 ± 0.58	4.01 ± 0.49	0.89	10.235	42	<0.001***
Willingness to communicate with peers	3.35 ± 0.62	4.15 ± 0.53	0.80	8.764	42	<0.001***
Public display willingness	2.56 ± 0.74	3.48 ± 0.66	0.92	8.143	42	<0.001***
Total score of willingness to communicate	2.98 ± 0.54	3.87 ± 0.46	0.89	11.568	42	<0.001***

***p < 0.001.

The changes of each dimension of the experimental group are shown in the radar chart in Figure 6. The outer polygon represents the posttest score, and the inner polygon represents the pretest score. The four dimensions have significantly expanded outward and the amplitude is balanced, indicating that the intervention has a stable promoting effect on all levels of communication willingness.

**Figure 6:**
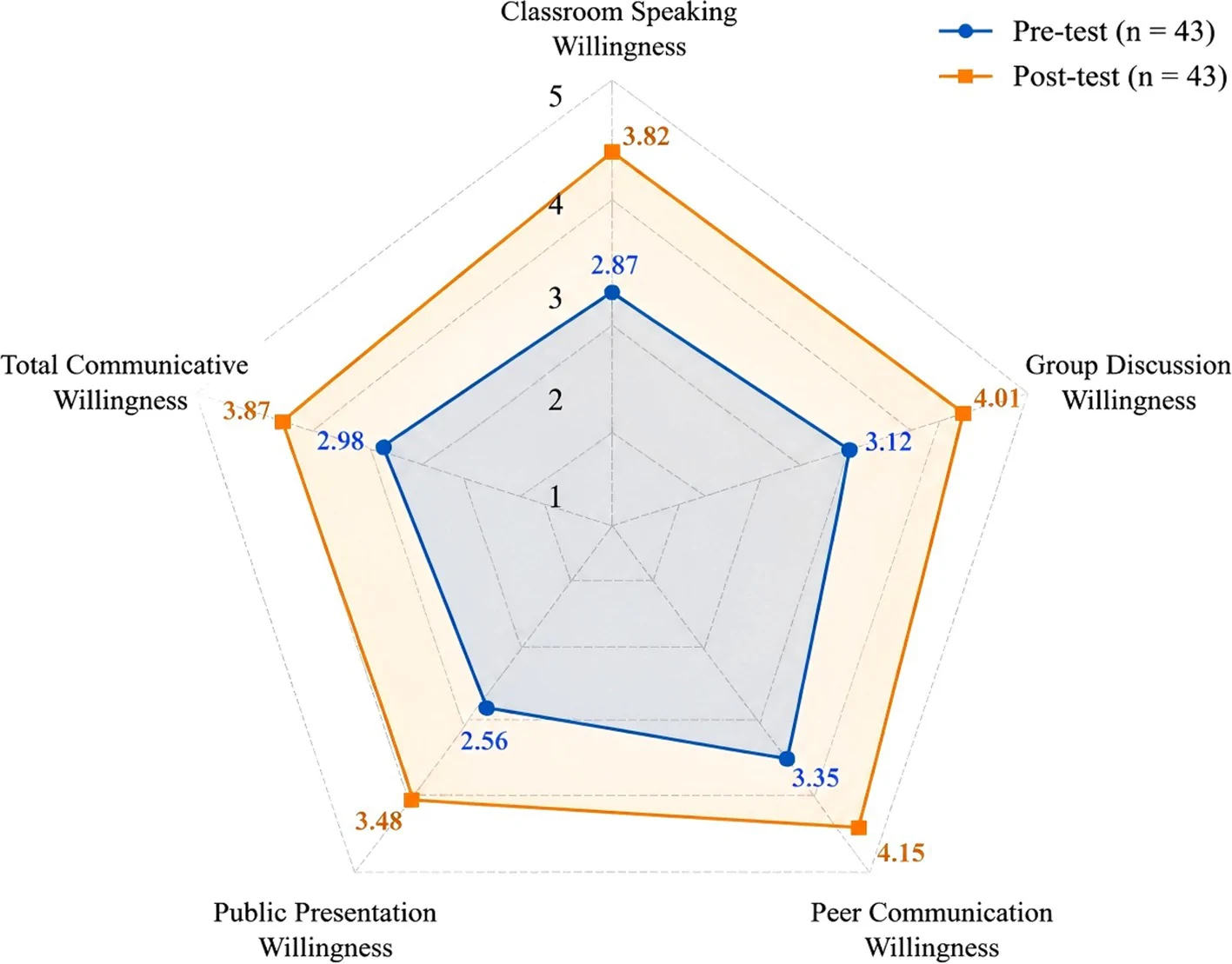
Radar chart of the changes of each dimension of communicative intention in the experimental group before and after the test.

The control group also showed within-group increases (Table 8), including a total-score gain of 0.34 points, from 2.97 to 3.31 (reported *t* = 4.106, df = 42, *p* < 0.001). Figures 6,7 display the respective sub-dimension changes. Descriptively, the experimental group’s total-score gain exceeded the control group’s gain by 0.55 points and was approximately 2.6 times as large. However, separate paired t-tests do not test whether these gains differ significantly between groups. H1b is therefore descriptively consistent with the reported pattern but is not directly established by the reported inferential analyses. Overall, H1 receives qualified support through H1a, while the between-group gain component remains untested.

**Table 8:** Paired sample *t*-test before and after the test of communicative intention in the control group.

Dimension	Pre-test m ± SD	Post-test m ± SD	Mean difference	*t* value	df	*p*-value
Willingness to speak in class	2.91 ± 0.71	3.24 ± 0.65	0.33	3.215	42	0.003**
Willingness to discuss in groups	3.08 ± 0.63	3.42 ± 0.58	0.34	3.578	42	0.001**
Willingness to communicate with peers	3.29 ± 0.67	3.67 ± 0.61	0.38	3.892	42	<0.001***
Public display willingness	2.61 ± 0.69	2.89 ± 0.72	0.28	2.437	42	0.019*
Total score of willingness to communicate	2.97 ± 0.57	3.31 ± 0.52	0.34	4.106	42	<0.001***

*p < 0.05, **p < 0.01, ***p < 0.001.

**Figure 7:**
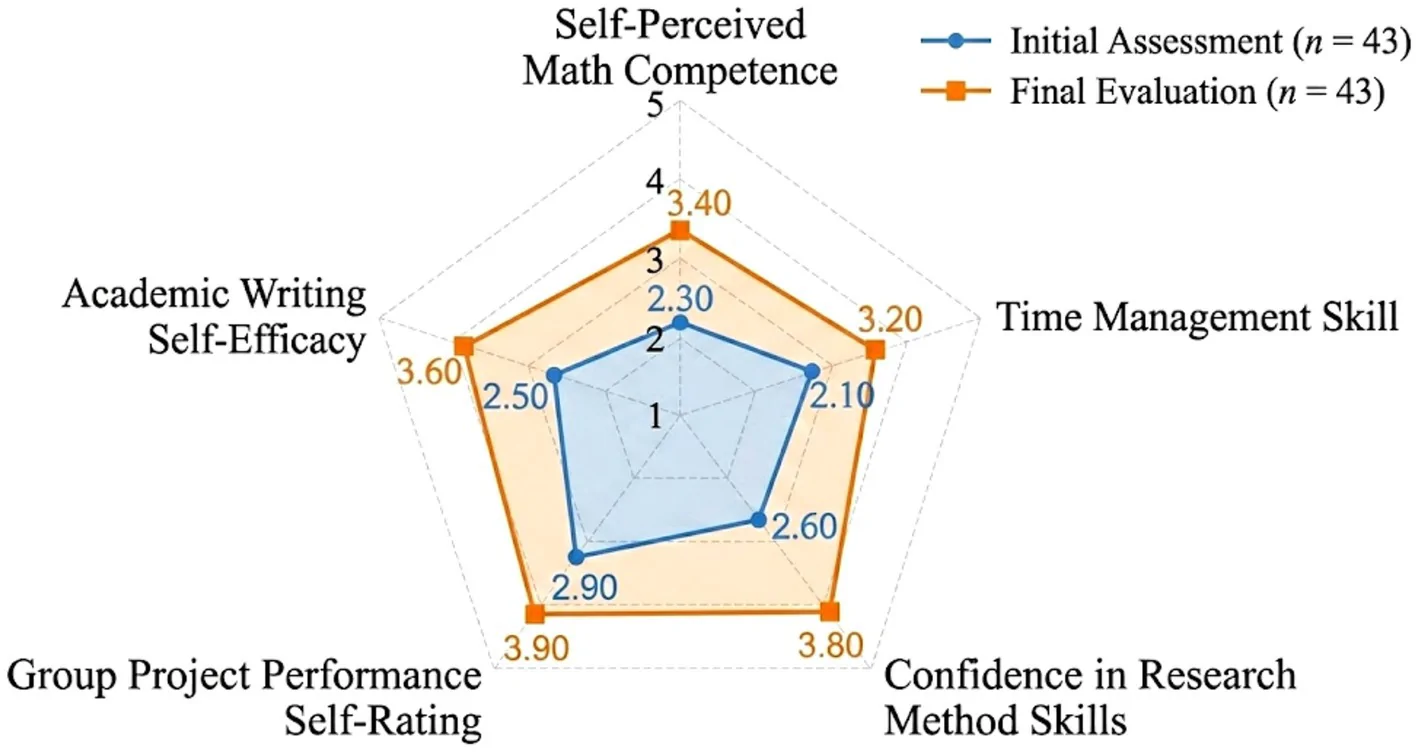
Radar chart of the pre-test and post-test changes of each dimension of communicative intention in the control group.

### 4.2 H2: correlations between cultural dimensions and WTC

#### 4.2.1 H2a to H2c zero-order associations

Pearson correlations were calculated using the experimental group’s post-test data (*n* = 43; Table 9). In hypothesis order, cultural cognition was positively correlated with WTC (H2a: *r* = 0.514, *p* < 0.001), cultural expression was positively correlated with WTC (H2b: *r* = 0.678, *p* < 0.001), and cultural identity was positively correlated with WTC (H2c: *r* = 0.596, *p* < 0.001). H2a-H2c, and hence H2, are supported by the reported correlations. These are zero-order associations and do not control for overlap among the cultural dimensions. The conditional associations required by H3 are examined separately in Section 4.3.

**Table 9:** Correlation matrix between dimensions of national cultural framework and willingness to communicate.

Variable	1	2	3	4
1. Cultural Cognition	1			
2. Cultural expression	0.623***	1		
3. Cultural identity	0.587***	0.701***	1	
4. Willingness to communicate	0.514***	0.678***	0.596***	1

***p < 0.001, *n* = 43.

In order to intuitively show the relationship between cultural expression and communicative intention, a scatter diagram of the two is drawn, as shown in Figure 8. Each point in the figure represents a student’s score, and the data points show an obvious distribution trend from bottom left to top right, which directly reflects the positive linear relationship between the score of cultural expression and the total score of communicative intention.

**Figure 8:**
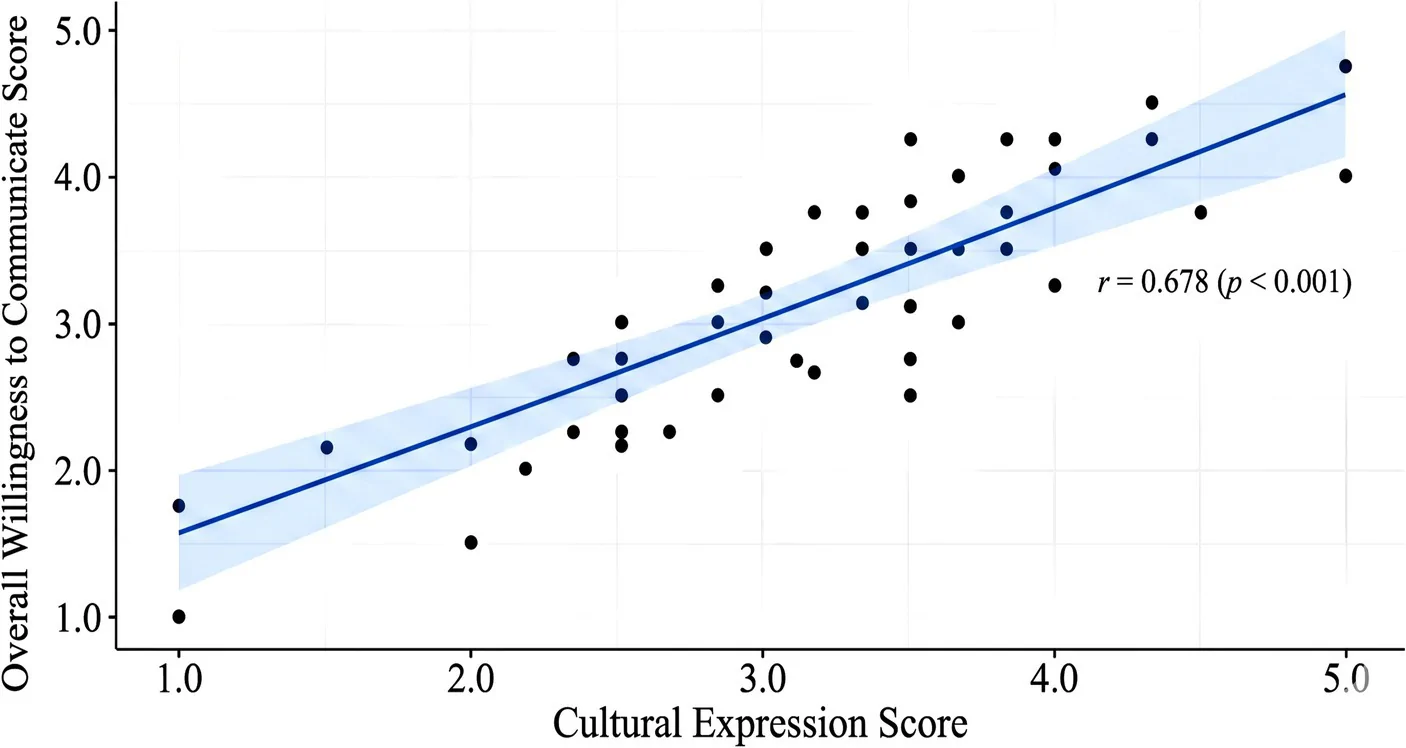
Total scatter points of cultural expression scores and communication willingness.

#### 4.2.2 H3: unique predictive contributions of cultural dimensions

#### 4.2.3 Simultaneous regression model

A simultaneous multiple regression used post-test WTC as the dependent variable and entered cultural cognition, cultural expression and cultural identity together, using the experimental group’s post-test data (*n* = 43). Table 10 reports the model and individual coefficients. The reported model explained 54.6% of the variance in WTC (*R*^2^ = 0.546; reported overall *p* < 0.001). Model-level fit describes the predictors jointly; H3a-H3c must be evaluated from their respective partial coefficients. These estimates concern associations at the same measurement occasion and do not identify causal or mediation pathways.

**Table 10:** Simultaneous regression of post-test WTC on the three cultural dimensions.

Predictor	B	SE	*β*	*t*	*p*	Tolerance
constant	1.235	0.342	—	3.611	0.001	—
Cultural cognition	0.124	0.087	0.153	1.425	0.162	0.582
Cultural expression	0.358	0.104	0.412	3.442	0.001**	0.467
cultural identity	0.247	0.096	0.295	2.573	0.014*	0.508

*p < 0.05, **p < 0.01; Overall model: *R*^2^ = 0.546, adjusted *R*^2^ = 0.511, *f* = 15.237, *p* < 0.001.

B denotes the unstandardized coefficient; SE, its standard error; and β, the standardized coefficient. The final column reports tolerance. All coefficients are from the same simultaneous model and are interpreted conditional on the other two predictors.

#### 4.2.4 H3a: cultural cognition

Cultural cognition had a positive but non-significant partial coefficient (*B* = 0.124, SE = 0.087, *β* = 0.153, *t* = 1.425, *p* = 0.162). H3a is not supported by this model. This result differs from the positive zero-order correlation supporting H2a because the regression conditions on cultural expression and cultural identity. It neither establishes the absence of any role for cultural knowledge nor demonstrates an indirect effect through the other dimensions.

#### 4.2.5 H3b: cultural expression

Cultural expression had a significant positive partial coefficient (*B* = 0.358, SE = 0.104, *β* = 0.412, *t* = 3.442, *p* = 0.001). This supports the positive unique-association component of H3b. Its standardized coefficient was numerically the largest of the three (0.412 versus 0.295 and 0.153). However, no formal coefficient-difference test was reported, so the comparative expectation that cultural expression is statistically the strongest predictor remains untested. H3b is therefore supported for its positive contribution, with its comparative component supported descriptively only.

#### 4.2.6 H3c: cultural identity

Cultural identity also had a significant positive partial coefficient (*B* = 0.247, SE = 0.096, *β* = 0.295, *t* = 2.573, *p* = 0.014). H3c is supported by the reported model after controlling for cultural cognition and cultural expression. Together, the dimension-specific results provide partial support for H3: cultural expression and cultural identity retain positive associations with WTC, whereas the cultural-cognition coefficient is not statistically significant. This pattern should not be interpreted as a tested sequence from cognition to expression or identity and then to WTC.

#### 4.2.7 Summary of hypothesis evaluations

Table 11 summarizes the evidence in the order H1, H2 and H3. It distinguishes supported associations, descriptive consistency and expectations that were not directly tested. Supplementary performance, observation and interview findings follow in Section 4.4 and are not used to substitute for the statistical tests required by these hypotheses.

**Table 11:** Summary of hypothesis evaluations in H1 to H3 order.

Hypothesis	Corresponding evidence	Evaluation
H1	Post-test group differences support H1a; gains are 0.89 versus 0.34, without a direct between-group gain test.	Qualified support
H1a	Post-test total: *t* = 5.324, *p* < 0.001, d = 1.15; all four sub-dimensions: *p* < 0.001.	Supported
H1b	Descriptive gain difference = 0.55 points; gain ratio ≈ 2.6. Separate paired tests do not test the between-group gain difference.	Descriptively consistent; not directly tested
H2	All three zero-order correlations with WTC are positive (*p* < 0.001).	Supported
H2a	Cultural cognition: *r* = 0.514, *p* < 0.001.	Supported
H2b	Cultural expression: *r* = 0.678, *p* < 0.001.	Supported
H2c	Cultural identity: *r* = 0.596, *p* < 0.001.	Supported
H3	Expression and identity have positive partial coefficients; cognition is non-significant.	Partially supported
H3a	Cultural cognition: *β* = 0.153, *p* = 0.162.	Not supported
H3b	Cultural expression: *β* = 0.412, *p* = 0.001; largest observed *β*, without a coefficient-difference test.	Positive contribution supported; comparative claim untested
H3c	Cultural identity: *β* = 0.295, *p* = 0.014.	Supported

### 4.3 Supplementary secondary outcomes and qualitative corroboration

#### 4.3.1 Secondary oral-performance outcomes and classroom observations

In addition to the core variable communicative intention, this study also examines the impact of teaching intervention on oral ability and classroom behavior. The independent sample t-test of the total score of the two groups’ oral posttest showed that the average oral performance of the experimental group was 72.56 ± 7.83, and that of the control group was 67.84 ± 8.21, which was significantly higher than that of the control group (*t* = 2.715, *p* = 0.008 < 0.01), indicating that the integration of culture into teaching not only improves the willingness to communicate, but also promotes the development of oral ability. The comparison results of each sub item dimension are shown in Table 12.

**Table 12:** Comparison of oral-task sub-scores between the experimental and control groups.

Scoring dimension	Experimental group M ± SD	Control group M ± SD	*t* value	*p*-value
Phonetic intonation	14.23 ± 2.15	13.67 ± 2.34	1.152	0.253
Lexical grammar	14.87 ± 2.08	13.95 ± 2.26	1.964	0.053
fluency	15.12 ± 1.96	13.42 ± 2.17	3.857	<0.001***
Content richness	16.34 ± 1.75	13.58 ± 2.03	6.823	<0.001***
Communicative appropriateness	13.00 ± 1.89	13.22 ± 1.94	−0.531	0.597

***p < 0.001.

The sub item results showed that the experimental group was significantly better than the control group in terms of fluency and content richness, and the difference in content richness was the largest. This shows that the theme of national culture provides sufficient expression materials for students, and significantly improves the amount of information and content depth of oral output; at the same time, familiar topics reduce the cognitive load and enable students to pay more attention to language expression, so the fluency is also significantly improved. There was no significant difference in pronunciation, intonation and communicative appropriateness between the two groups, indicating that cultural integration mainly affects oral performance through the content level, and has little effect on the pure voice level.

Classroom observations provided supplementary descriptive evidence (Figure 9). At week 16, the reported average frequency of students’ active speaking was 8.7 times per class in the experimental group and 5.2 in the control group; average group-discussion time was 22.3 and 15.8 min, respectively. Both groups showed increasing interaction over the intervention, with a visibly wider gap from approximately week 6. No inferential test of the difference in growth rates is reported, so these observations are treated as descriptive context rather than an additional test of H1 or H3.

**Figure 9:**
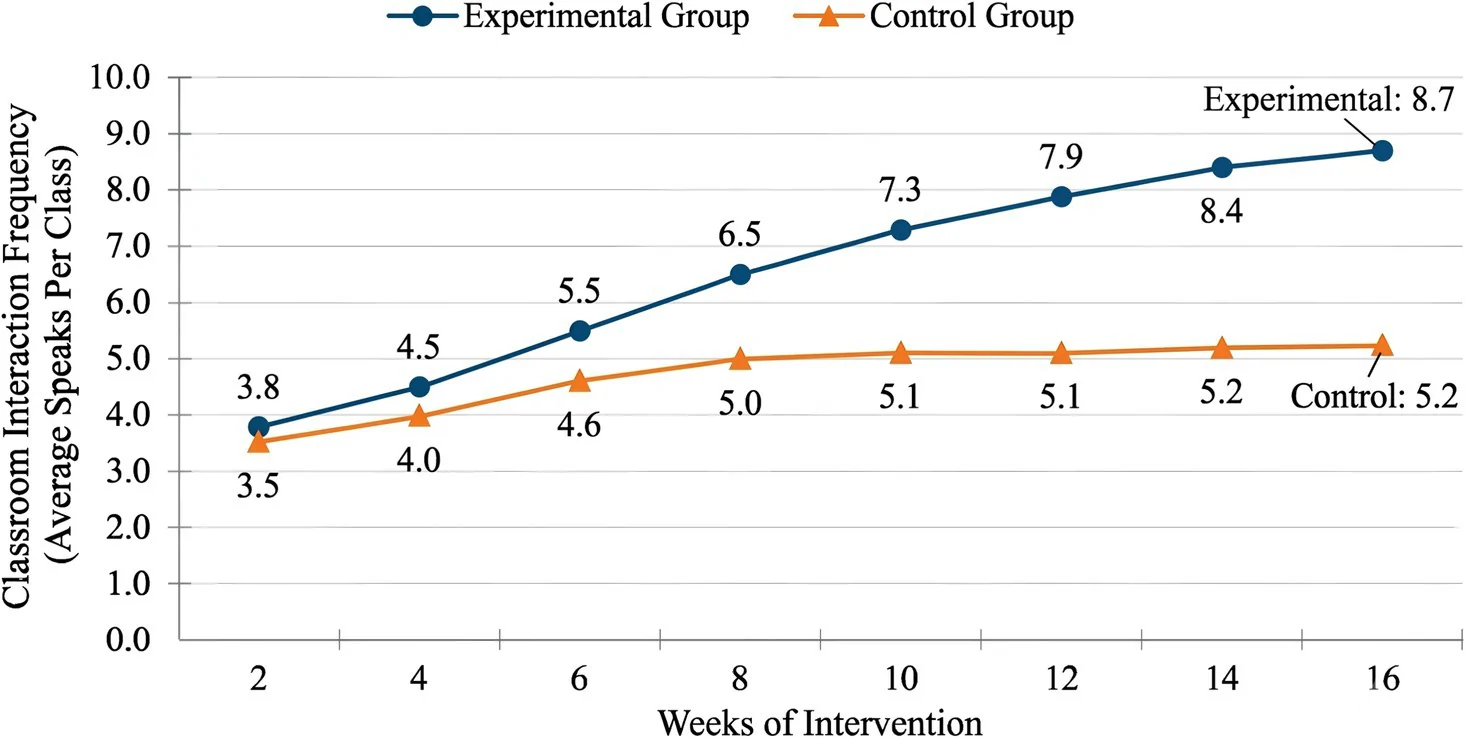
Broken line diagram of changes in classroom interaction frequency between the experimental group and the control group.

#### 4.3.2 Interview themes and qualitative corroboration

Through three-level coding analysis of the transcripts of the interviews of 12 students in the experimental group, four core themes were finally extracted. The frequency of reference points and typical expressions of each theme are shown in Table 13.

**Table 13:** Coding results for student interview themes.

Core theme	Frequency of reference point	Excerpts from typical statements
Topic familiarity reduces expression anxiety	10	“I know what to say when I talk about China. I am not afraid of making mistakes and making jokes when I talk about western topics.”
Cultural pride motivates expression	9	“Being able to speak your own culture in English has a special sense of achievement and wants to let others know the charm of Chinese culture
Content depth to enhance participation	8	“It is more meaningful to discuss cultural topics than daily topics, and I am willing to check the data and prepare in advance”
Practical value enhances learning motivation	7	“I can really use it when I meet foreigners in the future. I feel that I am not learning useless dumb English.”

The four interview themes concern topic familiarity and reduced expression anxiety, cultural pride and motivation to speak, content depth and preparation, and perceived practical value (Table 13). These accounts offer possible explanations for greater engagement and WTC. They provide qualitative corroboration and interpretive context, rather than independent statistical verification of H1-H3. Anxiety, self-efficacy and intrinsic motivation were not entered as measured mediators in the reported regression model, and interviews with experimental-group students alone cannot establish a causal pathway or a between-group difference in experience.

## 5 Discussion

### 5.1 Interpretation of the hypothesis-linked findings

The hypothesis evaluations are consolidated in Table 11 at the end of Section 4.3. H1a is supported by the reported post-test comparisons, whereas H1b is descriptively consistent with the gain pattern but lacks a direct between-group change test. H2a-H2c are supported by the reported zero-order correlations. For H3, the cultural-cognition coefficient is non-significant, while cultural expression and cultural identity have significant positive partial coefficients; the comparative component of H3b has not been formally tested. This distinction keeps the discussion aligned with the evidence presented for each hypothesis.

The differing conclusions for H2a and H3a are compatible because they answer different questions. H2a concerns the bivariate relationship between cultural cognition and WTC; H3a concerns its unique contribution after conditioning on the other two cultural dimensions. The loss of statistical significance in the latter analysis does not by itself identify mediation. It suggests that the role of cultural cognition requires further study with designs and measures that can distinguish shared variance, temporal ordering and potential indirect effects.

### 5.2 Layered comparison discussion

#### 5.2.1 The gain effect of communicative intention

The experimental group had a higher post-test WTC total score than the control group, with a reported between-group effect size of Cohen’s *d* = 1.15. Its mean pretest-to-posttest gain was 0.89 points, compared with 0.34 points in the control group, giving a descriptive gain ratio of approximately 2.6. This ratio is separate from the post-test effect size and does not establish a statistically significant between-group difference in change. The pattern suggests potential value in combining flipped classroom instruction with national cultural content, subject to the design and analytical limitations.

In the past, the research on flipped classroom oral teaching generally focused on the technical optimization of teaching process, and the core focus was on pure language skills indicators such as voice, fluency and complexity, forming a clear research bias of “emphasizing skills and ignoring culture”. Based on the complete preservation of the advantages of flipped classroom structure, this study systematically introduces the national cultural framework from the content level, and deeply combines “teaching form innovation” with “teaching content optimization”, achieving a significant increase in the amount of intervention effect. The innovative value of this discovery is that it proves that the effectiveness of the teaching model depends not only on the process design, but also on the psychological adaptation of the content itself; Local cultural content can inject internal driving force into the flipped classroom and break through the bottleneck of the effect of pure technology optimization.

#### 5.2.2 Hierarchical differences in the role of dimensions

The positive zero-order association between cultural cognition and WTC supports attention to cultural knowledge, but its non-significant partial coefficient indicates that its unique contribution was not established after controlling for expression and identity. Cultural expression and cultural identity retained positive partial associations. These findings suggest that knowledge, perceived expressive capability and cultural identification should be distinguished in teaching design and further research. They do not establish that knowledge must operate through a chain of expression and identity. In addition, cultural expression had the largest observed standardized coefficient, but its statistical superiority over the other coefficients remains untested.

#### 5.2.3 Systematic interpretation of mechanism of action

The interview themes in Section 4.4.2 suggest that familiar cultural content may reduce expression anxiety, that successful cultural expression may strengthen perceived capability, and that cultural pride and practical relevance may encourage motivation. These are plausible interpretations considered alongside the correlational and regression findings. They have not been verified as mediation effects: the reported model contains the three cultural dimensions and WTC, rather than measured anxiety, self-efficacy and intrinsic-motivation mediators. Longitudinal measurement and formal indirect-effect analyses would be needed to evaluate such pathways. Accordingly, H3 concerns conditional predictive associations, while these mechanisms remain theoretical explanations for future testing.

#### 5.2.4 Synergy of teaching and learning: constructing the integrated teaching paradigm of “form + content”

In previous studies, flipped classroom research and cultural integration research are basically in two parallel development tracks: flipped classroom research focuses on the innovation of teaching forms, and cultural integration research focuses on the reform of teaching content. The cross empirical research of the two is very rare. Most of the teaching practice is either to add cultural content in the traditional lecture class, which is difficult to carry out adequate language expression training due to the limitation of class hours, and finally alienated into cultural class; Either the flipped classroom uses the original teaching materials, fails to explore the value of cultural education, and the interaction is often a mere formality. In this study, the two are deeply integrated to achieve complementary advantages: the input link before class in flipped classroom solves the bottleneck of cultural knowledge, and the interaction link in class provides a sufficient platform for cultural expression practice; The content of national culture improves the interactive depth and sense of learning significance of flipped classroom, and solves the common problems of “interaction is superficial and students’ participation motivation is insufficient” in flipped classroom.

### 5.3 Research limitations

This study also has some limitations. Firstly, the sample is from a single local comprehensive university, and the sample size is relatively limited. The popularization of the research conclusion in key universities, higher vocational colleges, foreign language colleges and other different types of colleges and universities needs to be further verified. Secondly, given that intact natural classes were assigned to experimental and control conditions rather than random individual assignment, our intervention unit lies at the class level, whereas our statistical analyses treat individual students as independent observations. This may bring risks of pseudo replication. Multilevel modelling would be the ideal solution, yet it cannot yield stable estimates given only two class clusters in the present study. Future work should recruit more classroom clusters and adopt multilevel modelling. Thirdly, qualitative semi-structured interviews were only conducted with participants from the experimental group. Without interview data from the control group, our thematic interpretations may suffer from confirmation bias. Fourthly, the experimental period is 16 weeks, which is to investigate the effect of short-term intervention. Participants in the experimental group were exposed to novel cultural-integrated learning activities, so Hawthorne-type novelty effects may partly contribute to improved WTC outcomes, and long-term maintenance effect need to be tracked for a longer period. Finally, this study did not investigate the moderating effect of individual variables such as basic English level and personality traits on the intervention effect, and the benefit degree of students with different characteristics may be different. Future research can further expand the sample range and carry out multi-center empirical research; Long term follow-up can be carried out to separate novelty effects from genuine content-driven changes; control-group interview data are also recommended for future mixed-methods designs; we can also explore different intervention strategies for different groups of learners to achieve more accurate teaching design.

Additional analytical limitations follow from the distinction between hypotheses and reported tests. The paired t-tests do not directly establish the between-group gain difference specified by H1b, and the ranking of standardized regression coefficients does not formally test the comparative component of H3b. The H2 and H3 analyses use concurrent post-test scores from only 43 experimental-group students, limiting temporal and causal interpretation. Future studies should test group-by-time effects, specify coefficient comparisons in advance, and measure proposed mediators at appropriate time points before evaluating indirect effects.

## 6 Conclusion

In this 16-week quasi-experiment, the experimental group showed higher post-test WTC than the conventional flipped-classroom control group, supporting H1a in the reported analyses. Mean total-score gains were 0.89 and 0.34 points, respectively; the larger experimental-group gain is descriptive because a direct between-group change test was not reported. All three cultural dimensions were positively correlated with WTC, supporting H2. In the simultaneous regression, cultural expression (*β* = 0.412, *p* = 0.001) and cultural identity (*β* = 0.295, *p* = 0.014) retained positive associations, whereas cultural cognition did not reach statistical significance (*β* = 0.153, *p* = 0.162), providing partial support for H3. Cultural expression had the largest observed standardized coefficient, but the comparative expectation in H3b remains formally untested. Secondary oral-performance results and classroom observations provide additional context.

Interview themes suggest reduced anxiety, stronger perceived capability and greater motivation as possible explanations for the observed pattern. These themes do not demonstrate mediation or a complete causal chain. The findings offer preliminary support for integrating national cultural content with flipped oral-English instruction, while the two-class design, concurrent predictor measurements and limited sample constrain causal and generalizable conclusions. Further studies with more classes, longitudinal measures and explicit tests of change and indirect effects are needed to evaluate the proposed benefits and mechanisms.

## Data Availability

The raw data supporting the conclusions of this article will be made available by the authors, without undue reservation.
