# Supplementary material for: The impact of national cultural framework on college students’ willingness to communicate in oral English tasks in flipped classroom mode: a quasi experimental study
Source: Front Psychol. 2026 Oct 7;17:1910813. doi: 10.3389/fpsyg.2026.1910813 (PMC13683701; doi:10.3389/fpsyg.2026.1910813)
Supplement: Supplementary file 1 [file Data_Sheet_1.docx]

**Supplementary Materials (Appendix)**

Note: 5‑point Likert scale for both scales: 1 = Strongly Disagree; 2 = Disagree; 3 = Neutral; 4 = Agree; 5 = Strongly Agree

**Appendix A. Revised L2 Willingness to Communicate Scale**

Dimension 1: Willingness to speak in class (5 items)

1. I am willing to volunteer answers when the teacher asks questions in English class.

2. I am willing to express my own viewpoints actively in whole‑class English discussions.

3.I dare to raise questions in English even when I am not fully sure about my answers.

4. I am willing to give short spontaneous responses when the teacher invites me to speak.

5. I would like to voice my opinions even if other classmates remain silent.

Dimension 2: Willingness to discuss in groups (4 items)

6. I am willing to take an active part in small‑group English discussions.

7. I tend to initiate turns to speak during group‑work oral tasks.

8. I am ready to exchange different ideas with group members in English.

9. I will keep contributing to group discussion rather than just listening passively.

Dimension 3: Willingness to communicate with peers (5 items)

10. I am willing to chat with my classmates in English after class.

11. I would like to cooperate with partners to finish oral role‑play tasks.

12. I am happy to give oral feedback to my classmates in English.

13. I will try to keep English conversations going with my peers.

14. I feel comfortable communicating with classmates in English on daily topics.

Dimension 4: Public display willingness (4 items)

15. I am willing to make oral presentations in front of the whole class.

16. I dare to show my oral output results publicly in English‑class activities.

17. I am ready to answer questions from audiences after my class presentation.

18. I hope to get more chances to give public oral performances in English.

**Appendix B. Self‑developed National Cultural Framework Scale (21 items)**

Dimension1: Cultural Cognition (7 items)

1. I have a good command of basic knowledge of Chinese traditional historical stories.

2. I can recognize typical Chinese cultural symbols and their cultural connotations.

3. I am familiar with the customs behind major Chinese traditional festivals.

4. I understand the core values embodied in traditional Chinese culture.

5. I know basic knowledge of Chinese intangible cultural heritage.

6. I can distinguish the cultural meanings of representative Chinese art forms.

7. I have accumulated adequate background knowledge of Chinese folk culture.

Dimension2: Cultural Expression (7 items)

8. I can translate Chinese cultural proper nouns accurately into English.

9. I am confident in explaining Chinese cultural phenomena in English.

10. I can adopt proper expression strategies when introducing Chinese culture to foreigners.

11. I am able to make cross‑cultural comparisons between Chinese and foreign cultures in English.

12. I can organize my language logically when telling Chinese stories in English.

13. I know how to make up appropriate expressions when facing culturally‑loaded words I do not know.

14. I feel capable of completing oral tasks themed on Chinese culture in English.

Dimension 3: Cultural Identity (7 items)

15. I feel proud of the splendid traditions of my national culture.

16. I have a strong sense of responsibility to inherit Chinese excellent culture.

17. I am willing to introduce Chinese culture to people from other countries.

18. I recognize the unique value and charm of Chinese local culture.

19. I hope to spread Chinese cultural content through English communication.

20. I hold positive emotional attitudes toward our national cultural traditions.

21. I think it is meaningful for college students to tell Chinese stories in English.

**Appendix C. Semi‑structured Interview Protocol**

1. What is your experience of doing oral tasks integrated with Chinese national culture in flipped‑classroom learning?

2. In what ways has your willingness to speak English changed during this 16‑week course? What do you think brings about such changes?

3. Compared with general textbook‑based oral topics, how do Chinese‑culture‑themed tasks influence your anxiety, confidence and motivation for oral English output?

4. What are the strengths and weaknesses of this national‑culture‑integrated flipped‑classroom oral teaching?

5. What suggestions would you give to improve oral English teaching combining national cultural content?
